# Analogue of dynamic Hall effect in cavity magnon polariton system and coherently controlled logic device

**DOI:** 10.1038/s41467-019-11021-2

**Published:** 2019-07-03

**Authors:** J. W. Rao, S. Kaur, B. M. Yao, E. R. J. Edwards, Y. T. Zhao, Xiaolong Fan, Desheng Xue, T. J. Silva, Y. S. Gui, C.-M. Hu

**Affiliations:** 1 Department of Physics and Astronomy, University of Manitoba, Winnipeg, R3T 2N2 Canada; 2 The Key Lab for Magnetism and Magnetic Materials of Ministry of Education, Lanzhou University, Lanzhou, 730000 People’s Republic of China; 3 State Key Laboratory of Infrared Physics, Shanghai Institute of Technical Physics, Chinese Academy of Sciences, Shanghai, 200083 People’s Republic of China; 4 Quantum Electromagnetics Division, National Institute of Standards and Technology, Boulder, CO 80305 USA

**Keywords:** Ferromagnetism, Spintronics

## Abstract

Cavity magnon polaritons are mixed quasiparticles that arise from the strong coupling between cavity photons and quantized magnons. Combining high-speed photons with long-coherence-time magnons, such polaritons promise to be a potential candidate for quantum information processing. For harnessing coherent information contained in spatially distributed polariton states, it is highly desirable to manipulate cavity magnon polaritons in a two-dimensional system. Here, we demonstrate that tunable cavity magnon polariton transport can be achieved by strongly coupling magnons to microwave photons in a cross-cavity. An analog to the dynamic Hall effect has been demonstrated in a planar cavity spintronic device, where the propagation of cavity-magnon-polaritons is deflected transversally due to hybrid magnon-photon dynamics. Implementing this device as a Michelson-type interferometer using the coherent nature of the dynamic Hall and longitudinal signals, we have developed a proof-of-principle logic device to control the amplitude of cavity-magnon-polaritons by encoding the input microwave phase.

## Abstract (web summary)

Exploring photon-polariton interactions advances not only the understanding of polariton dynamics but also the modern technologies. Here the authors take advantage of strong coupled magnons and microwave photons in a cross-cavity to achieve tunable cavity magnon polariton transport which can be potentially applied as logic devices.

## Introduction

Strong light-matter interactions between different physical platforms not only enable the study of fundamental polariton physics, but also could advance modern technologies, such as quantum information processing^1–4^. Recently, a number of pioneering works have shown that by coupling magnons (collective spin excitations in a crystal lattice) with cavity photons, the constructed cavity-magnon polariton (CMP)^5–7^ possess the advantages of flexible tunablity as well as long-coherence time^5,6,8–15^. So far these works are mainly restricted to the frequency/time domain, while the manipulation of polaritons in real space remains relatively unexplored. To further advance coherent information processing by utilizing CMPs, a device with three fundamental functions of switching, transport, and basic logic operations^16^ is required. Inspired by resonator lattices allowing on-chip photon flow control^17^ and cavity grid electrodynamics allowing scalable quantum computation^18^, two-dimensional periodic arrays of planar resonators play an important role, especially when they are coupled to the two-level system (TLS) to achieve the goal of manipulating spatially separated bosonic fields. In such an application-oriented array, it is highly important to have the ability to switch on/off the information stream as well as to eliminate the noisy cross talk between two neighboring cross-bar resonator unit cells^19,20^. In this study, cavity-magnon-polariton dynamics based on these unit cells are shown to fulfill the basic requirements for information processing, providing promising platform for future CMP-based computation techniques.

Here in this work, we form a cross-cavity (termed an X-cavity in the following text) by using two identical orthogonal half-wavelength microstrip line resonators. By using strong coupling effects between the X-cavity and magnons to generate X-CMPs, polariton transport with directional-selection features, accompanied with three non-degenerate polariton modes in spectrum, can be clearly revealed. Based on the similarities between X-CMP transport and the Hall effect, a concise dynamic Hall tensor has been put forward that describes the experimental results precisely. Through the Hall-like transport in X-CMP dynamics, a logic gate implementing Boolean functions is realized by controlling the phase of the input microwave. Our work demonstrates the possibility for controlling cavity-magnon-polariton flow in an on-chip device, showing the potential for selectively transferring multimode coherent information with good coherence and long lifetime. By combining the above advantages, we present an on-chip phase-controlled X-CMP logic gate, showing potential for CMP-based information processing. Moreover, our X-CMP device provides unique properties distinct from traditional electrical logic devices. Inherited from the photon component, X-CMPs can achieve high speeds (comparable with light speed) in transport^16,21^. In addition, based on linear harmonic dynamics^13^ and passive media, our X-CMP logic device exhibits the advantage of reduced power requirement. In this manner, the revealed functionality of X-CMPs could provide a platform for research as well as forming a building block for future CMP-based computation techniques, paving the way to develop polariton-based devices for advancing coherent information processing technologies.

## Results

### Construction of X-CMP

**Fig. 1:**
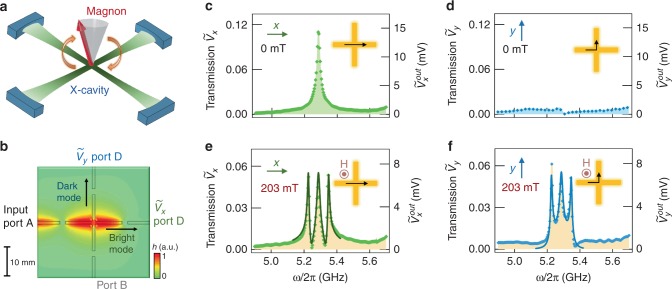
Construction of X-CMP. **a** Schematic figure of a magnon coupled to a cross-cavity. **b** Simulated microwave field distribution for the fundamental mode showing that microwave propagation is confined along the *x*-direction when the microwaves are sent through port A, with scale bar showing the dimension and the color scale showing the normalized amplitude of microwave magnetic field (*h*). **c** *x*-direction and **d** *y*-direction microwave transmission spectra (left axis) as well as output voltage (right axis) measured at 0 mT. **e** *x*-direction and **f** *y*-direction microwave transmission spectra (left axis) as well as output voltage (right axis) measured at 203 mT, solid lines shows the calculated results from an analog to the dynamic Hall model

For manipulating the propagation of coherent information, we designed a planar X-cavity that couples to a magnon mode, shown in Fig. 1a. Microwave photons are confined in an X-cavity with identical geometries in two orthogonal directions, in which minimal coupling between the two orthogonal boson fields in the two arms can be obtained. The length of each orthogonal arm is 20 mm. By introducing a 1-mm diameter YIG sphere to the X-cavity, such a hybrid system enables magnons to interact with different boson fields, and therefore leads to the manipulation of a spatially distributed polariton flow.

In order to construct the hybrid magnon–photon states, it is beneficial to simulate the electromagnetic field distribution first. By applying input microwaves at port A, the X-cavity mode in the *x*-direction is directly excited and is regarded as a bright mode^22^. It can also be found that the cavity mode in the *y*-direction is more isolated from the incoming microwave excitation, showing the typical behavior of a dark mode. Such bright-dark mode characteristics can be further seen in transmission measurements using a vector network analyzer (VNA). Normalized to the input AC voltage of 0.13 V, a resonant peak $\tilde V_x$ can be observed from the bright mode (see Fig. 1c), while the dark mode spectrum $\tilde V_y$ shows almost no signal (Fig. 1d). Since our device works in the linear dynamics regime, similar results can also be observed for lower input powers.

By creating a cavity-magnon polariton in the X-cavity (X-CMP), a dynamic voltage $\tilde V_y$ can be established across the bright mode cavity, producing a measurable signal along the *y*-direction. This coupled state is produced by placing a YIG sphere in the intersection of the X-cavity, maximizing the overlap coefficient between the magnon and cavity modes. An external magnetic field is applied perpendicular to the cavity plane to excite and tune the magnon mode. With the near-uniform microwave field considered^23^, fundamental magnon mode ($\omega _m$) is excited dominantly in small YIG sphere. In addition to the fundamental magnon mode $\omega _m$, we also notice the weak excitation of non-uniform magnon modes, which, however, does not affect the performance of the X-CMP since their resonant frequencies are significantly different from the fundamental magnon mode. As $\omega _m$ is tuned to match the cavity mode ($\omega _c$), the polariton flow is deflected to the y-direction, providing the enhanced $\tilde V_y$ seen in Fig. 1f. This ability to change the direction of polariton flow reveals a degree of freedom for controlling polariton transport in two-dimensional scales. In addition, the on/off switching rate of this system could be further optimized to microsecond scales by adopting fast magnetic field control systems using pulsed magnetic fields^24^ with typical field-sweep rate of 10^3^–10^5^ T s^−1^, and to nanosecond scales by phase control using high-speed phase shifters^25^.

Besides the transport manipulation abilities, our X-CMP brings multi-channel dynamics for information processing, through its unique cavity-magnon-polariton spectrum. In contrast to the typical two-level splitting gap seen in conventional cavity-magnon-polariton systems, here we obtain three hybrid modes from X-CMP dynamics (see Fig. 1e, f), with one central mode and two side peaks. In the following sections, we demonstrate that the central mode, regarded as an emerged freedom in spectrum, has different dynamics compared with two side polariton peaks. Moreover, different from bare cavity mode which only supports the photon dynamics, here the central mode is a result of magnon–photon coupling effects, allowing the mode to be controlled by the static magnetic field. A degree of freedom in the frequency domain can be further incorporated into our X-CMP through the flexible control of the polariton flow in real space, leading to a functional on-chip device for basic logic operations. However, realizing such applications requires an understanding of X-CMP transport as well as the correlation between different polariton modes; in the next part, these are achieved by introducing an analog to the dynamic Hall effect in an X-cavity.

### Transport: analogy of dynamic Hall effect

**Fig. 2:**
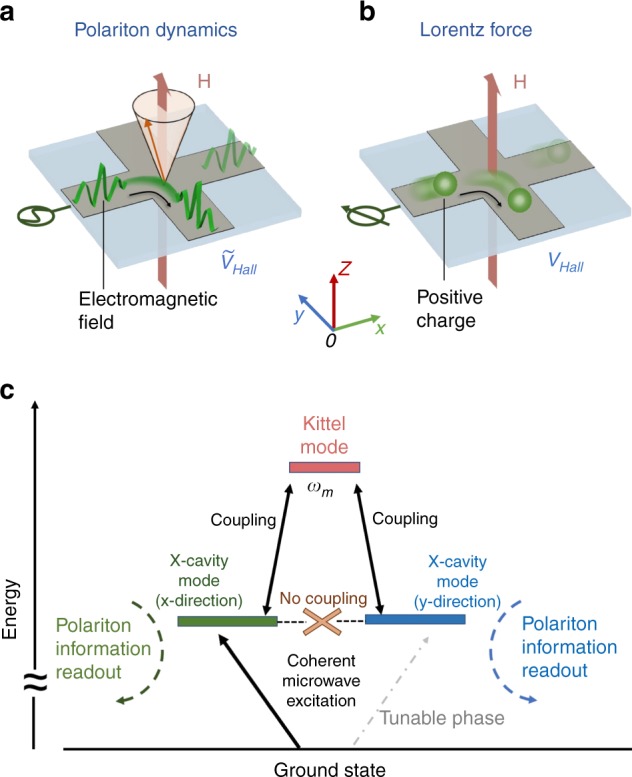
Drawing analogy between X-CMP transport and Hall effect. **a** Schematic diagram of the dynamic Hall effect. Construction of X-CMP dynamics leads to the deflection of polariton flow from the *x*-direction toward the *y*-direction. **b** Schematic diagram of the Hall effect. A charge current flows along the *x*-direction. The Lorentz force acts on the charge, producing a static Hall signal. In both (**a**, **b**) the external applied static magnetic field **H** is along the *z-*direction, perpendicular to the sample plane. **c** A description of the coupling between a magnon and the X-cavity. The energy diagram illustrates the frequencies of the degenerate cavity modes $\omega _c$ as well as the magnon mode $\omega _m$. The magnon couples to the X-cavity modes with a coupling strength of ${\mathrm{\Omega }}_0$

The observed deflection of polariton flow in X-CMP dynamics can be explained by drawing an analogy between strongly coupled magnon–photon dynamics and the Hall effect^26^. The built-in polariton flow in the transverse direction of the X-cavity, as shown in the schematic diagram in Fig. 2a, is similar to the Hall effect (shown in Fig. 2b) where a transverse Hall signal is generated due to the deflection of current caused by the Lorentz force^27^. This similarity can be further expressed through mathematics. The Hall effect^27^ can be generally described by the equation ${\mathbf{V}} = \hat R{\mathbf{I}}$, with $\hat R$ representing the Hall resistance tensor^27–29^. Analogously, we will show in this section that polariton transport in our X-CMP is governed by a transfer tensor $\hat T$. As an external magnetic field *H* is applied to satisfy the condition $\omega _c \approx \omega _m$, off-diagonal components in $\hat T$ become significant, giving rise to a measurable Hall-like signal in the transverse direction. Thus, we establish a dynamic Hall-like effect in our X-CMP system.

After a qualitative comparison, here we quantitatively analyze X-CMP transport. Based on the coupling scheme between the X-cavity and a magnon mode described in Fig. 2c, the Hamiltonian of the system can be expressed as,

1 $$\begin{array}{*{20}{l}} H \hfill & = \hfill & {\hbar \omega _ca_x^{\mathrm{\dagger }}a_x + \hbar \omega _mm^{\mathrm{\dagger }}m + \hbar \omega _ca_y^{\mathrm{\dagger }}a_y} \hfill \\ {} \hfill & {} \hfill & { + \hbar {\mathrm{\Omega }}_0(m^{\mathrm{\dagger }}a_x + ma_x^{\mathrm{\dagger }}) + \hbar {\mathrm{\Omega }}_0(m^{\mathrm{\dagger }}a_y + ma_y^{\mathrm{\dagger }})} \hfill \end{array}$$

where $a_x(a_x^{\mathrm{\dagger }})$, $a_y(a_y^{\mathrm{\dagger }})$ are the annihilation (creation) operators of the X-cavity modes in the *x*- and *y*-directions. $m(m^{\mathrm{\dagger }})$ is the annihilation (creation) operator of the magnon mode, and ${\mathrm{\Omega }}_0$ is the coupling strength between the magnon and an individual cavity mode. Further, using input–output theory and considering photon annihilation (creation) as representing a photon bath, we can calculate the transmission of our X-CMP to be (see details in Supplementary Notes 1 and 2),

2 $$\left( {\begin{array}{*{20}{c}} {\tilde V_x^{{\mathrm{out}}}} \\ {\tilde V_y^{{\mathrm{out}}}} \end{array}} \right) = 2\kappa _p\frac{{\hat T}}{{{\it{det}}(\hat T)}}\left( {\begin{array}{*{20}{c}} {\tilde V_x^{{\mathrm{in}}}} \\ {\tilde V_y^{{\mathrm{in}}}} \end{array}} \right)$$

where $\tilde V_x^{{\mathrm{in}}}$ ($\tilde V_y^{{\mathrm{in}}}$) and $\tilde V_x^{{\mathrm{out}}}$ ($\tilde V_y^{{\mathrm{out}}}$) are defined as the input and output signals along the *x*- and *y*-directions, respectively. For the simplicity of transmission normalization, here we first set $\left( {\tilde V_x^{{\mathrm{in}}},\tilde V_y^{{\mathrm{in}}}} \right)^T = (1,0)^T$. $\kappa _p$ is the coupling strength between the feed lines and the X-cavity. Importantly, the tensor $$\hat T = \left( {\begin{array}{*{20}{c}} {T_{xx}} & {T_{xy}} \\ {T_{yx}} & {T_{yy}} \end{array}} \right)$$ governs the direction of polariton flow and can be written as the following,

3 $$\begin{array}{*{20}{l}} {T_{xx} = T_{yy} = A + {\mathrm{\Omega }}_0^2/B} \hfill \\ {T_{xy} = T_{yx} = - {\mathrm{\Omega }}_0^2/B} \hfill \end{array}$$

where the parameters *A* and *B* are associated with the cavity mode and the magnon mode, and written as $A = i(\omega - \omega _c) - \beta \omega _c$ and $B = i(\omega - \omega _m) - \alpha \omega _m$, respectively. $\beta = 0.0017$ and $\alpha = 0.0001$ are the damping rates of the cavity and magnon mode.

With this model established, we can quantitatively explain the polariton transport revealed in Fig. 1. The external *H* field adjusts the detuning of the magnon mode from cavity resonance, leading to a direct control of diagonal and off-diagonal magnitudes. At $\mu _0H = 0$, the diagonal terms of $\hat T$ are dominant, causing a resonant peak to appear in the longitudinal signal $\tilde V_x$; while the off-diagonal terms tend to be zero with $|{\mathrm{\Omega }}_0^2| \ll |B|$, producing the weak background in $\tilde V_y$. From the cavity profile in Fig. 1c we determine $\kappa _p$ to be 3.3 MHz. Tuning to the zero detuning condition with *μ*_0_*H* = 203 mT leads to $|{\mathrm{\Omega }}_0^2|$ and |*B*| having comparable magnitudes and consequently non-zero off-diagonal terms in $\hat T$. At this condition, a Hall-like voltage $\tilde V_y$ can be observed, as shown in Fig. 1f.

Having demonstrated the control of a Hall-like voltage through the tensor $\hat T$, we further analyze the coherent energy conversion between magnons and photons in our X-CMP. Coherent coupling effects are represented by the polariton peaks in the spectrum at zero detuning. These three polariton modes indicate the generation of quasiparticles as well as effective energy transfer between the X-cavity modes and the magnon mode. Quantitatively, the three non-degenerate resonances in Fig. 1e and f can be identified as $\omega = \omega _c$ and $\omega = \omega _c \pm \sqrt 2 {\mathrm{\Omega }}_0$, simply by setting the denominator ${\it{det}}(\hat T)$ in Eq. (2) to be zero (see details in Supplementary Note 3). The separation of the two side peaks thus corresponds to $2\sqrt 2 {\mathrm{\Omega }}_0$, allowing us to extract the coupling strength ${\mathrm{\Omega }}_0$/2*π* = 42 ± 2 MHz from the measured separation in Fig. 1e and f. It can be found that this coupling strength exceeds the dissipation rates of the magnon mode ($\alpha \omega _m = 0.6\,{\mathrm{MHz}}$) and the photon mode ($\beta \omega _c = 9\,{\mathrm{MHz}}$), showing our X-CMP system operates in the strong coupling regime. With this measured coupling strength, we can well reproduce $\tilde V_x$ and $\tilde V_y$ with theoretical results (solid lines) from our Hall-like transport model. The good agreement between our experimental and theoretical findings constitutes a playground for further engineering of the X-CMP dynamics.

### Multi-channel dynamics: X-CMP dispersion

Here we clarify the multi-channel dynamics behind the three non-degenerate modes. First, the appearance of the central polariton mode can be explained by taking the bright-dark mode behavior into account. Despite the fact that the vertical X-cavity mode is a dark mode at *μ*_0_*H* = 0, it is excited by applying *μ*_0_*H* toward the zero detuning condition. As a consequence, the microwave modes in the X-cavity can be either in-phase $(a_x + a_y)/\sqrt 2$ or out-of-phase $(a_x - a_y)/\sqrt 2$, analogous to the case of two magnon modes coupled with a cavity mode^13^.

For the side (in-phase) modes, the coherent magnon–photon interaction results in a collective enhancement of the polariton gap by a factor of $\sqrt 2$, so that the gap between side modes is $2\sqrt 2 {\mathrm{\Omega }}_0$. While for the central (out-of-phase) mode, the coupling effects from the two cavity modes on the magnon mode cancel, resulting in zero net coupling strength and the reappearance of the original cavity mode in the center of the spectrum.

**Fig. 3:**
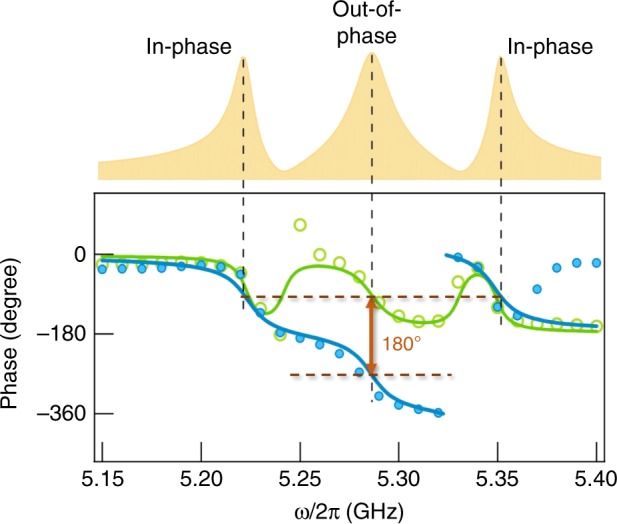
Phase of X-CMP dynamics. The measured phases of X-CMP dynamics in $\tilde V_x$ and $\tilde V_y$ have been plotted, displayed in green circles and blue circles, respectively. Here the phase in *y*-direction has been wrapped. By using the Hall-like transport model, these phases can be well explained. We show the calculated phases for both *x*- and *y*-directions with green solid line and blue solid line, respectively. A schematic X-CMP profile has been plotted above the figure to show the three non-degenerate polariton modes. We demonstrate that the two side modes are in-phase while the central mode shows out-of-phase behavior

In addition, the in-phase and out-of-phase behavior of our X-CMP can be further verified by measurements, shown by the $\tilde V_x$ and $\tilde V_y$ phase spectra in Fig. 3. We can clearly see in the experimental data that the two side peaks show in-phase dynamics. The central mode in the *x*- and *y*-directions has a 180° phase lag and therefore displays a typical out-of-phase behavior. Together with the measured results, we also plot the calculated phase spectra using Eq. (2). As shown in Fig. 3, at resonant conditions, our theoretical model reproduces the key experimental features of in-phase and out-of-phase behavior between the central and side modes. At off-resonant conditions, the parasitic signal shown in Fig. 1f may become dominant, leading to disagreement between theoretical phase spectra and measured ones.

**Fig. 4:**
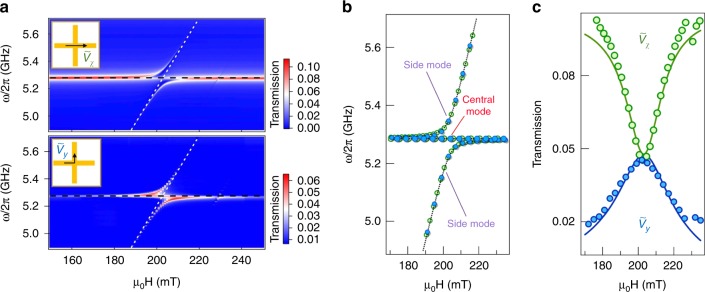
X-CMP dispersion. **a** Amplitude mappings of $\tilde V_x$ and $\tilde V_y$ plotted as a function of applied magnetic field measured for the configurations shown in the inset. White dashed lines indicate the expected magnon dispersion according to Kittel’s formula and the black dashed lines show the cavity frequency. **b** Dispersion of the hybridized modes. **c** Resonance feature of the longitudinal signal $\tilde V_x$ and the Hall signal $\tilde V_y$ near $\omega _m = \omega _c$, where the central mode is at $\omega = \omega _c$. In (**b**, **c**), symbols are experimental data deduced from (**a**) and the dashed or solid lines are the calculated results using Eq. (2)

Second, the measured $\tilde V_x$ and $\tilde V_y$ spectra as the external magnetic field *μ*_0_*H* is adjusted are plotted in Fig. 4a. Between the anti-crossing polariton modes, a central polariton mode can be clearly observed in both the *x*- and *y*-directions. Such dispersion can be explained by the dynamic Hall tensor. Calculated results are plotted as dashed lines in Fig. 4b, together with the extracted polariton mode positions from the measured mappings. Since $\tilde V_x$ and $\tilde V_y$ share the same denominator ${\it{det}}(\hat T)$, we find identical dispersions in the *x*- and *y*-directions in both the measurements and calculations.

However, despite their identical dispersions, the amplitude evolutions of these modes show distinctly different behaviors when reading from the *x*- and *y*-directions. Theoretically, $\tilde V_x$ and $\tilde V_y$ are determined by the terms *T*_*xx*_ and *T*_*yx*_, respectively, and therefore show different dependences on the term ${\mathrm{\Omega }}_0^2$/*B*. This leads to the fact that the amplitudes of $\tilde V_x$ and $\tilde V_y$ show opposite behaviors as we drive $\omega _m$. As an example, we plot the measured amplitudes of the central mode as a function of *μ*_0_*H* in Fig. 4c with solid circles. The opposite amplitude evolution of $\tilde V_x$ and $\tilde V_y$ is not only revealed by measurements but is also well explained theoretically by the model in Eq. (2), as indicated by the solid lines.

It is worth noting that the central polariton mode readout in the *y*-direction enables a special method for observing a strongly coupled magnon–photon signal. Conventionally in the evolution of CMP modes, when detuned away from zero, the cavity mode signal is dominant since it is the bridge that connects the CMP with the external photon bath. However, in X-CMP dynamics, as the Hall-like signal $\tilde V_y$ is built on a dark cavity mode and is mathematically determined by *T*_*yx*_, the contribution of the cavity mode vanishes. In this manner, detuning away from zero in X-CMP systems can result in the polariton signal reaching zero amplitude, which may be utilized to eliminate coherent magnon–photon signals in information processing.

### Polaritonic logic gate

**Fig. 5:**
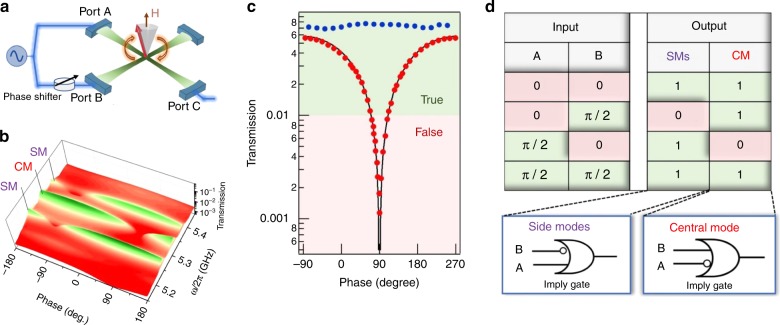
Imply gate logic operations based on dynamic Hall-like transport. **a** Experimental setup using a low-loss mechanical phase shifter to introduce the phase difference ${\mathrm{\Phi }}$ between ports A and B. **b** Transmission amplitude mapping measured at port C by continuously tuning the phase difference between the two inputs. The central mode (CM) amplitude shows a different phase dependence compared with the side modes (SMs). **c** Transmission amplitude of the central mode plotted as a function of the phase. Here, the blue solid symbols represent the result measured at 0 mT. **d** The logic table for the imply gates constructed using the response of the side modes (SMs) and central mode (CM)

Based on the demonstration of on/off polariton switching as well as multi-channel dynamic behavior in our X-CMP system, in this section, these features are combined together to demonstrate basic logic operations in X-CMP transport. To study these coherent coupling induced logic operation, an X-cavity was configured as a Michelson-type interferometer^30–32^. Figure 5a shows a schematic diagram of the experimental setup. A microwave source was split into two coherent parts and injected into the device from ports A and B, respectively. Two microwave signals with different phases couple with the YIG sphere in the X-cavity, forming a X-CMP interferometer. Tuning the phase difference ${\mathrm{\Phi }}$ using a mechanical phase shifter, one can control the interference between the two X-CMP beams precisely. In order to properly understand the coherence of the X-CMP dynamics, the phase correlation of the magnetization precession relative to the polarization of the microwave magnetic fields according to the Polder tensor should be considered.

Setting *μ*_0_*H* = 203 mT (zero detuning condition), a pronounced interference pattern appears, as shown in Fig. 5b. With ${\mathrm{\Phi }} \approx 90^\circ$, the central mode signals from both arms interfere destructively, suppressing the signal measured at port C. When ${\mathrm{\Phi }}$ is adjusted to −90° the signals constructively interfere producing a maximal signal. The side modes exhibit opposite dynamic behaviors, with destructive interference occuring at ${\mathrm{\Phi }}$ ≈ −90° and constructive interference at ${\mathrm{\Phi }}$ ≈ 90°. The 180° phase shift in the interference pattern between the central and side modes further confirms the phase coherence of the hybridized modes shown in Fig. 3.

The amplitudes of the central mode in Fig. 5b are plotted as a function of phase in Fig. 5c using red symbols. To quantitatively interpret these curves, the dynamical Hall tensor must be taken into account. With the tensor $\hat T$, the output signal at port C can be expressed as (see details in Supplementary Note 3)

4 $$\tilde V_C = \sqrt 2 \kappa _p(T_{xx} + ie^{i{\mathrm{\Phi }}}T_{xy})\tilde V^{{\mathrm{in}}}/{\it{det}}(\hat T)$$

where $\tilde V^{{\mathrm{in}}}$ is the microwave input signal from the microwave generator. This theoretical equation clearly reveals the phase ${\mathrm{\Phi }}$ dependence of the output voltage and reproduces the measurements well (solid lines in Fig. 5c). In comparison, the transmission amplitudes measured at 0 mT are plotted as blue solid symbols, which are not sensitive to the phase ${\mathrm{\Phi }}$ because of the negligible dynamic Hall effect ($T_{xy} \sim 0$).

Due to the high dynamical range of these signals, one can classify them into regions of true and false to create a logic device based on whether the side modes or the central mode is present. Figure 5d shows the logic table for the imply gates which can be constructed in this way for the side modes and central mode. For the side modes, the imply gate returns true (or 1) if the microwave phase at port A is greater than or equal to the microwave phase at port B; whereas for the central mode, the imply gate returns true if the microwave phase at port B is greater than or equal to the microwave phase at port A. This experimental effect clearly shows the possibility of implementing logic gates using the dynamic Hall effect in a strongly coupled cavity-magnon–photon device.

In summary, we have demonstrated a dynamic Hall-like effect for polariton signals in a planar X-cavity spintronic device that utilizes strong cavity photon-magnon coupling to manipulate the direction of polariton flow. The dispersion, amplitude variation and phase correlation between the different hybridized modes can be explained through our model utilizing a the dynamic Hall tensor. The excellent coherence of the cavity-magnon-polariton enables the implementation of logic concepts based on wave interference, which may lead to room temperature polaritonic devices and the possibility of exploiting polaritons for quantum computation and simulation.

## Methods

### Device description

The cross-cavity with planar dimensions 40 × 40 mm was fabricated on a very low-loss laminate substrate with a dielectric constant of 2.20 ± 0.02 over a wide frequency range. The thickness of the substrate was 1.57 ± 0.05 mm with a copper thickness of 17.5 μm on both sides. The fabricated cavity includes two identical orthogonal half-wavelength microstrip line resonators that have a length and width of 20 and 1 mm, respectively. The coupling gap between the feed lines and the resonator was 2.0 mm. This value controls the quality factor of the cavity and has been optimized using electromagnetic simulations.

A 1 mm diameter YIG sphere is located in the intersection of the cross-cavity. The YIG magnon mode follows the Kittel dispersion equation $\omega _m = \gamma \mu _0(H + H_a)$, where *μ*_0_ is the vacuum permeability, *γ* = 2*π* × 26.0 GHz T^−1^ is the gyromagnetic ratio and *μ*_0_*H*_*a*_ = 3.0 mT is the anisotropy field of the YIG sphere. The static magnetic field *H* is applied perpendicularly to the plane of the cross-cavity.

### Measurement setup

The frequency spectra are measured using a vector network analyzer with an input power of −5 dBm. Through a feedline port, microwave transmission is characterized, and the external coupling strength between the feedline port and the X-cavity is measured to be $\kappa _p = 3.3\,{\mathrm{MHz}}$. In the section demonstrating polaritonic logic device, the microwave are divided into two paths to excite the X-cavity with a relative phase delay. By inserting a phase shift into one path, the quantity of the phase delay can be precisely tuned.

### Theory

Details about the theory of X-CMP transport are provided in Supplementary Notes 1, 2, and 3.

## Supplementary information

Supplementary information

## Data Availability

The data that support the findings of this study are available from the corresponding authors upon reasonable request.
