# Supplementary material for: Analogue of dynamic Hall effect in cavity magnon polariton system and coherently controlled logic device
Source: Nat Commun. 2019 Jul 3;10:2934. doi: 10.1038/s41467-019-11021-2 (PMC6610622; doi:10.1038/s41467-019-11021-2)
Supplement: Supplementary file 1 — Supplementary information [file 41467_2019_11021_MOESM1_ESM.pdf]

Supplementary Information

Analogue of dynamic Hall effect in cavity magnon polariton system and coherently controlled logic device

Rao et al.

This PDF file includes:

Supplementary Note 1: Theoretical description of magnon-photon coupling in x-cavity

Supplementary Note 2: Input-output relations

Supplementary Note 3: Transmission of X-CMP dynamics

Supplementary References

Supplementary Note 1. Theoretical description of magnon-photon coupling in x-cavity

Hamiltonian of X-CMP dynamics

The whole system can be viewed as a coupled cavity magnon system in a photon bath. The Hamiltonian of the whole system has the following form:

$$H = H_{sys} + H_{bath} + H_{int} \tag{1}$$

Here, H_{sys} is the Hamiltonian of the coupled cavity magnon system isolated with surroundings, and H_{bath} is the Hamiltonian of the photon bath. As for the H_{int}, it represents the interaction effect between the coupled cavity magnon system and the photon bath. The detailed expressions of these three parts are shown as following:

$$H_{sys} = \hbar\omega_c a_x^\dagger a_x + \hbar\omega_m m^\dagger m + \hbar\omega_c a_y^\dagger a_y + \hbar\Omega_0(m^\dagger a_x + m a_x^\dagger) + \hbar\Omega_0(m^\dagger a_y + m a_y^\dagger) \tag{2}$$

$$H_{bath} = \int \hbar\omega_k \sum_{r=A}^{D}(p_{k,r}^\dagger p_{k,r} + \frac{1}{2})dk \tag{3}$$

$$H_{int} = \int i\hbar\lambda[(p_{k,A}a_x^\dagger - p_{k,A}^\dagger a_x) + (p_{k,C}a_x^\dagger - p_{k,C}^\dagger a_x) + (p_{k,B}a_y^\dagger - p_{k,B}^\dagger a_y) + (p_{k,D}a_y^\dagger - p_{k,D}^\dagger a_y)]dk \tag{4}$$

$a_x(a_x^\dagger)$, $a_y(a_y^\dagger)$ represent the annihilation (creation) operators of X-cavity modes in x- and y- directions. $m(m^\dagger)$ is the annihilation (creation) operator of the magnon. $p_{k,r}(p_{k,r}^\dagger)$ is the annihilation (creation) operator of the photon with wave vector k, while the subscript index r=A,B,C,D corresponds to the port A,B,C,D respectively.

The equation motion for the extra-cavity photon

The equation of motion for the extra-cavity photon operator in Heisenberg representation reads

$$\frac{dp_{k,A}}{dt} = -\frac{i}{\hbar}[p_{k,A}, H] = -i\omega_k p_{k,A} - \lambda a_x \tag{5}$$

The solution of this dynamic equation can be formally written as

$$p_{k,A}(t) = e^{-i\omega_k(t-t_0)}p_{k,A}(t_0) - \lambda\int_{t_0}^{t} e^{-i\omega_k(t-t')}a_x(t')dt' \tag{6}$$

Analogously, we can also get the expressions of the photon operators for the other three ports:

$$p_{k,C}(t) = e^{-i\omega_k(t-t_0)}p_{k,C}(t_0) - \lambda\int_{t_0}^{t} e^{-i\omega_k(t-t')}a_x(t')dt' \tag{7}$$

$$p_{k,B}(t) = e^{-i\omega_k(t-t_0)}p_{k,B}(t_0) - \lambda\int_{t_0}^{t} e^{-i\omega_k(t-t')}a_y(t')dt' \tag{8}$$

$$p_{k,D}(t) = e^{-i\omega_k(t-t_0)}p_{k,D}(t_0) - \lambda\int_{t_0}^{t} e^{-i\omega_k(t-t')}a_y(t')dt' \tag{9}$$

The equation motion for the intra-cavity photon

Similar to Supplementary equation (5), we can write the equations of motion for the two orthogonal X-cavity modes in the Heisenberg representation.

$$\frac{da_x}{dt} = -\frac{i}{\hbar}[a_x, H_{sys}] + \int_0^\infty \lambda p_{k,A}dk + \int_0^\infty \lambda p_{k,C}dk \tag{10}$$

$$\frac{da_y}{dt} = -\frac{i}{\hbar}[a_y, H_{sys}] + \int_0^\infty \lambda p_{k,B}dk + \int_0^\infty \lambda p_{k,D}dk \tag{11}$$

By substituting H_{sys} and Supplementary equation (6)-(9) into Supplementary equation (10) and (11), we obtain a new form of equations of motion for x- and y- directions, respectively.

$$-i\omega a_x = -i\omega_c a_x - i\Omega_0 m + \sqrt{2\kappa_p}(p_A^{in} + p_C^{in}) - 2\kappa_p a_x \tag{12}$$
$$-i\omega a_y = -i\omega_c a_y - i\Omega_0 m + \sqrt{2\kappa_p}(p_B^{in} + p_D^{in}) - 2\kappa_p a_y \tag{13}$$

where κ_p is the coupling strength between feedlines and X-cavity, which follows the relation $\sqrt{\kappa_p} = \sqrt{\pi}\lambda$.

Supplementary Note 2. Input-output relations

The extra-cavity asymptotic output operators at $t = +\infty$ can be related to the input operators at $t_0 = -\infty$ and the cavity photon ones through a linear relationship[1,2]. The standard definitions of the input and output photons are $p_{k,r}^{in} = p_{k,r}(t_0)e^{i\omega_k(t_0)}$ and $p_{k,r}^{out} = p_{k,r}(t)e^{i\omega_k(t)}$. By substituting them into Supplementary equation (6)-(9), we can get the response formula of the photon with wave vector k at each port, shown below

$$p_{k,r}^{out} = p_{k,r}^{in} - \sqrt{2\pi}\lambda a(\omega_k) \tag{14}$$

Here, the $a(\omega_k)$ is the Fourier Transformation of $a_x(t')$ or $a_y(t')$ depending on the port number, r.

$$a(\omega_k) = \frac{1}{\sqrt{2\pi}}\int_{-\infty}^{\infty} e^{i\omega_k t'} a(t')dt' \tag{15}$$

The input and output signals are the sum of photons with different wave vectors i.e. the wave packets. Therefore, we define the input and output wave packets as

$$p_r^{in} = \frac{1}{\sqrt{2\pi}}\int_0^{\infty} e^{-i\omega_k t} p_{k,r}^{in} dk \tag{16}$$
$$p_r^{out} = \frac{1}{\sqrt{2\pi}}\int_0^{\infty} e^{-i\omega_k t} p_{k,r}^{out} dk \tag{17}$$

Substituting these two definitions into the Supplementary equation (14) and transferring $a_r(\omega_k)$ from the frequency domain back to the time domain $a(t)$, we can obtain the input-output relation as

$$p_r^{out} = p_r^{in} - \sqrt{2\pi}\lambda a(t) \tag{18}$$

Supplementary Note 3. Transmission of X-CMP dynamics

Start from the dynamic equation of magnon in Heisenberg representation

$$\begin{aligned}\frac{dm}{dt} &= -\frac{i}{\hbar}[m, H_{sys}] \\ &= -i\omega_m m - i\Omega_0(a_x + a_y)\end{aligned} \tag{19}$$

If we assume the solution of this equation follows the general form as $m = |m|e^{-i\omega t}$, then Supplementary equation (19) should be:

$$(\omega - \omega_m)m = \Omega_0(a_x + a_y) \tag{20}$$

Now, we put the intrinsic damping of the magnon into this equation i.e. $\omega_m \to \omega_m - i\alpha\omega_m$, then Supplementary equation (20) becomes

$$(\omega - \omega_m + i\alpha\omega_m)m = \Omega_0(a_x + a_y) \tag{21}$$

In experiment, for general assumption, the port A and port C are the input ports, and the port B and port D are output ports. Under this condition, we can set p_C^{in} and p_D^{in} as zero and obtain the equations of motion of the coupled cavity magnon system in the cross cavity as following:

$$
\begin{aligned}
&[\omega - \omega_c + i(\beta_{in}\omega_c + 2\kappa_p)]a_x - \Omega_0 m = i\sqrt{2\kappa_p}p_A^{in} \\
&[\omega - \omega_c + i(\beta_{in}\omega_c + 2\kappa_p)]a_y - \Omega_0 m = i\sqrt{2\kappa_p}p_B^{in} \\
&(\omega - \omega_m + i\alpha\omega_m)m - \Omega_0(a_x + a_y) = 0
\end{aligned}
\tag{22}
$$

Here, $\beta_{in}\omega_c$ is the intrinsic damping of the cavity. We can also use a lumped damping factor β to represent the loaded damping of the cavities, i.e. $\beta\omega_c = \beta_{in}\omega_c + 2\kappa_p$.

Dynamics hall effect in X-CMP

Firstly, if we only input microwave power at port A. As a result, input condition can be simplified as $p_A^{in} = \widetilde{V}_x^{in}$ and $p_B^{in} = 0$, using Supplementary equation (22), we get the expressions of a_x and a_y in the frequency domain as:

$$
\begin{aligned}
a_x &= -\frac{\sqrt{2\kappa_p}\widetilde{V}_x^{in}}{i(\omega-\omega_c) - \beta\omega_c + \frac{\Omega_0^2}{i(\omega-\omega_m)-\alpha\omega_m + \frac{\Omega_0^2}{i(\omega-\omega_c)-\beta\omega_c}}} \\
a_y &= a_x + \frac{\sqrt{2\kappa_p}\widetilde{V}_x^{in}}{i(\omega-\omega_c)-\beta\omega_c}
\end{aligned}
\tag{23}
$$

Substituting these two expressions into Supplementary equation (18), we get the output signals from the port C and port D, respectively, i.e. $p_C^{out} = \widetilde{V}_x^{out} = -\sqrt{2\kappa_p}a_x$ and $p_D^{out} = \widetilde{V}_y^{out} = -\sqrt{2\kappa_p}a_y$:

$$
\begin{aligned}
\widetilde{V}_x^{out} &= \frac{2\kappa_p\widetilde{V}_x^{in}}{i(\omega-\omega_c) - \beta\omega_c + \frac{\Omega_0^2}{i(\omega-\omega_m)-\alpha\omega_m + \frac{\Omega_0^2}{i(\omega-\omega_c)-\beta\omega_c}}} \\
\widetilde{V}_y^{out} &= \frac{2\kappa_p\widetilde{V}_x^{in}}{i(\omega-\omega_c) - \beta\omega_c + \frac{\Omega_0^2}{i(\omega-\omega_m)-\alpha\omega_m + \frac{\Omega_0^2}{i(\omega-\omega_c)-\beta\omega_c}}} - \frac{2\kappa_p\widetilde{V}_x^{in}}{i(\omega-\omega_c)-\beta\omega_c}
\end{aligned}
\tag{24}
$$

Because of the geometric symmetry of our device, it's straightforward to draw a conclusion that if signal was input from port B i.e. $p_A^{in} = 0$ and $p_B^{in} = \widetilde{V}_y^{in}$, we can also observe the normal transmission signal and the dynamic Hall signal from the port C and port D, respectively. By means of this symmetric property, we have transformed supplementary equation (24) into a matrix equation which is shown as:

$$
\frac{1}{2\kappa_p}\begin{bmatrix} i(\omega-\omega_c) - \beta\omega_c + \frac{\Omega_0^2}{i(\omega-\omega_m)-\alpha\omega_m} & \frac{\Omega_0^2}{i(\omega-\omega_m)-\alpha\omega_m} \\ \frac{\Omega_0^2}{i(\omega-\omega_m)-\alpha\omega_m} & i(\omega-\omega_c) - \beta\omega_c + \frac{\Omega_0^2}{i(\omega-\omega_m)-\alpha\omega_m} \end{bmatrix}\begin{bmatrix} \widetilde{V}_x^{out} \\ \widetilde{V}_y^{out} \end{bmatrix} = \begin{bmatrix} \widetilde{V}_x^{in} \\ \widetilde{V}_y^{in} \end{bmatrix}
\tag{25}
$$

To better describe the dynamic Hall effect, this matrix equation has been transformed into a much more straightforward form:

$$
\begin{pmatrix} \widetilde{V}_x^{out} \\ \widetilde{V}_y^{out} \end{pmatrix} = 2\kappa_p\frac{\hat{T}}{det(\hat{T})}\begin{pmatrix} \widetilde{V}_x^{in} \\ \widetilde{V}_y^{in} \end{pmatrix}
\tag{26}
$$

Here, $\hat{T}$ is the dynamic Hall tensor in X-CMP system, which plays an important role in controlling the direction of polariton flow. The detail of $\hat{T}$ is shown below as

$$
\hat{T} = \begin{bmatrix} i(\omega-\omega_c) - \beta\omega_c + \frac{\Omega_0^2}{i(\omega-\omega_m)-\alpha\omega_m} & -\frac{\Omega_0^2}{i(\omega-\omega_m)-\alpha\omega_m} \\ -\frac{\Omega_0^2}{i(\omega-\omega_m)-\alpha\omega_m} & i(\omega-\omega_c) - \beta\omega_c + \frac{\Omega_0^2}{i(\omega-\omega_m)-\alpha\omega_m} \end{bmatrix}
\tag{27}
$$

with the denominator $det(\hat{T})$ as,

$$\det(\hat{T}) = [i(\omega-\omega_c)-\beta\omega_c]^2 + \frac{2\Omega_0^2[i(\omega-\omega_c)-\beta\omega_c]}{i(\omega-\omega_m)-\alpha\omega_m} \tag{28}$$

Because of the non-zero off-diagonal terms of the transfer matrix, the Hall signal is generated in the experiment. And since these off-diagonal terms exhibit a resonance response of the external magnetic field, the dynamic Hall signal can be modulated by the external field. We have shown a detailed description of the dynamic Hall signal in the manuscript.

Two-port input experiment

The second step of our experiment is the Two-port input experiment. The input signal is $\widetilde{V}^{in}$, and we split it into two branches with same amplitude. A mechanical phase shifter was added into one branch to induce a phase difference between the two coherent signals (Φ). Then, these two signals were injected into the cross cavity from port A and port B, simultaneously. In theory, they can be described as:

$$\begin{aligned} p_A^{in} &= \widetilde{V}_x^{in} = \widetilde{V}^{in}/\sqrt{2} \\ p_B^{in} &= \widetilde{V}_y^{in} = ie^{i\Phi}\widetilde{V}^{in}/\sqrt{2} \end{aligned} \tag{29}$$

By substituting these two input signals into Supplementary equation (26), it's straightforward to obtain the output signal from port C:

$$\widetilde{V}_C = \sqrt{2}\kappa_p(T_{xx} + ie^{i\Phi}T_{xy})\widetilde{V}^{in}/det(\hat{T}) \tag{30}$$

And the spectra of the output signal in frequency domain is:

$$\widetilde{V}_C = \frac{\sqrt{2}\kappa_p\widetilde{V}^{in}}{i(\omega-\omega_c)-\beta\omega_c+\frac{(1+ie^{i\Phi})\Omega_0^2}{i(\omega-\omega_m)-\alpha\omega_m+\frac{(1-ie^{i\Phi})\Omega_0^2}{i(\omega-\omega_c)-\beta\omega_c}}} \tag{31}$$

Supplementary References

1. Ciuti, C., & Carusotto, I., Input-output theory of cavities in the ultrastrong coupling regime: The case of time-independent cavity parameters, *Phys. Rev. A* **74**, 033811 (2006).
2. Clerk, A. A., Devoret, M. H., Girvin, S. M., Marquardt, F. & Schoelkopf, R. J. Introduction to quantum noise, measurement, and amplification. *Rev. Mod. Phys.* **82**, 11551208 (2010).
